# Protocol: a Comprehensive Approach to Reduce Elderly functional decline in Diabetes: the CARED study

**DOI:** 10.1186/s12877-025-06673-3

**Published:** 2025-11-28

**Authors:** Alice Margherita Ornago, Francesca Remelli, Caterina Trevisan, Alberto Finazzi, Eugenia Casali, Diego Mastino, Efisio Cossu, Giovanna Manconi, Carla Serra, Alessandro Sestu, Gianluca Perseghin, Marcello Monesi, Stefano Volpato, Giuseppe Bellelli, Angelo Scuteri

**Affiliations:** 1 School of Medicine and Surgery, University of Milano-Bicocca, Piazza dell’Ateneo Nuovo 1, Milan, Italy; 2 Aging Research Center, Department of Neurobiology, Care Sciences and Society, Karolinska Institutet and Stockholm University, Stockholm, Sweden; 3 Department of Medical Sciences, University of Ferrara, Ferrara, Italy; 4 Diabetes Unit, Policlinico di Cagliari, Cagliari, Italy; 5 Internal Medicine Unit, Policlinico di Cagliari, Cagliari, Italy; 6 Department of Medical Sciences and Public Health, University of Cagliari, Cagliari, Italy; 7 Metabolic Medicine Unit, Policlinico di Monza, Monza, Italy; 8 Territorial Diabetes Unit, AUSL of Ferrara, Ferrara, Italy; 9 Acute Geriatric Unit, IRCCS San Gerardo Foundation, Monza, Italy

**Keywords:** Diabetes, Comprehensive geriatric assessment, Short physical performance battery

## Abstract

**Background:**

Type 2 diabetes mellitus (T2DM) is a common chronic disease in older adults and a leading cause of death and disability worldwide. While recent diabetes guidelines introduce the Comprehensive Geriatric Assessment (CGA) as a key tool for managing diabetes treatments, evidence of its effectiveness in delaying functional impairment and achieving patient-reported outcomes (PROs) remains lacking. This article describes the protocol of a randomized parallel-group controlled trial aimed at evaluating the effect of a CGA-guided plan of care on physical performance changes over time as compared with a usual-care group.

**Methods:**

Three centers will participate in this study, each recruiting 60 older adults with T2DM from their outpatient clinics. Eligible patients will be randomized into either the usual-care or intervention group in a 1:1 allocation ratio. All patients will receive standard care according to current diabetes guidelines and undergo CGA. For the intervention group, the CGA will guide a multidimensional intervention. Follow-up visits will be scheduled at 6 and 12 months. The primary outcome will be the change in physical performance at the end of the follow-up, as assessed by the Short Physical Performance Battery score, with a comparison between the control and intervention groups.

**Discussion:**

From this study, we will contribute to the growing evidence of the impact of CGA on the assessment and management of older patients with T2DM and its complications. Results will be disseminated through a peer-reviewed journal and presentations at conferences. Final results will be shared with a broader audience through collaborations with patients with diabetes and their caregiver association at local and national levels. The findings will inform the extent to which a CGA-driven care plan may significantly reduce functional impairment and improve patient and caregiver satisfaction.

**Trial registration:**

ClinicalTrials.gov ID: NCT06842459 – registered February 24, 2025 – retrospectively registered.

**Supplementary Information:**

The online version contains supplementary material available at 10.1186/s12877-025-06673-3.

## Background

Type 2 diabetes mellitus (T2DM) is a chronic disease characterized by hyperglycemia, usually due to insulin resistance and progressive loss of insulin secretion or function from the pancreatic β-cells. Globally, the prevalence of T2DM gradually increases with age, exceeding 20% in people over 65 and rising to 25% among those aged 75–79 years [1–3]; indeed, most of the patients with T2DM is 65 and older.

T2DM represents a substantial burden to the healthcare system worldwide [1]. In older patients, it is associated with an increased risk of developing complications due to both macrovascular (e.g., myocardial infarction, stroke, peripheral arterial disease) and microvascular (e.g., retinopathy, nephropathy, and neuropathy) damages [4, 5]. These factors can contribute to multimorbidity and multiple drug prescriptions, leading to an increased risk of adverse clinical events, poor quality of life, and functional loss [5, 6]. Specifically, older patients with T2DM tend to develop disability 6 to 7 years earlier, spend one to two more years with disability, and experience higher rates of premature death (4.6 years earlier) compared to non-diabetic individuals of the same age and sex [7].

In light of these considerations, older patients with T2DM require a comprehensive clinical approach that accounts for multiple variables and outcomes, including relevant patient-reported outcomes (PROs) [8]. Incorporating patient-reported outcome measures (PROMs) and patient-reported experience measures (PREMs) into clinical practice may facilitate identifying patient’s unmet needs and monitoring their evolution over time while improving communication and participation of patients and/or caregivers.

The Comprehensive Geriatric Assessment (CGA) is a validated and effective method for assessing and managing the complex care needs of older patients through goal-driven interventions [9]. Its multidimensional approach, encompassing medical diagnoses, pharmacotherapies, cognitive and physical performance, and environmental and social issues, is essential for developing individualized care plans based on PROMs and PREMs.

The American Diabetes Association (ADA) has recently incorporated elements of the CGA into the standard care of older patients with T2DM, recommending its use to guide therapeutic approaches in T2DM management [10]. However, as these recommendations are still in the early stage of implementation and have not yet become an integral part of routine clinical practice, there is currently no solid evidence supporting the effectiveness of CGA and its associated interventions in delaying functional impairments or achieving key PROs in older adults with T2DM. Our study will address this gap by integrating these outcomes, as part of PROMs, into the care goals of older patients with T2DM.

## Methods/design

### Aims

The primary aim of this study is to evaluate the impact of a CGA-based care planning intervention on physical performance changes of older patients with T2DM, as assessed by the Short Physical Performance Battery (SPPB) [11]. As secondary aims, we will assess the effectiveness of the intervention in reducing the incidence of adverse health outcomes, including falls, sarcopenia, disability, cognitive decline, depression, emergency department admission, hospitalization, institutionalization, and death.

### Study design

This study consists of a multicenter randomized parallel-group controlled trial that will take place at the diabetology outpatient clinics of the University of Cagliari, the University of Ferrara, and the Policlinico di Monza, Italy. Recruitment of the study participants will start in January 2025.

The study protocol was planned in conformity with the Standard Protocol Items: Recommendations for Interventional Trials (SPIRIT) 2025 statement ( Supplementary Table 1) [12].

### Patient screening

All patients attending the diabetology outpatient clinics will be consecutively screened for potential participation in the study.

### Inclusion/exclusion criteria

Eligibility will be determined according to the inclusion and exclusion criteria reported in Table 1.

**Table 1:** Exclusion and inclusion criteria

Inclusion criteria	Exclusion criteria
• Age 75 + years • Diagnosis of T2DM • SPPB ≥ 4, indicating none to moderate functional impairment • Ability to understand and speak Italian • Willingness to participate in the study	• Current institutionalization or residence outside the hospital’s catchment area at enrolment • Severe disability, defined as dependence on 3 or more Activities of Daily Living (ADL) • Cognitive impairment or dementia, defined as a MoCA adjusted score < 17.36/30 (ES = 0) • Diagnosis of schizophrenia, bipolar disorder or other psychotic disorders • History of cancer requiring treatment in the past three years, excluding non-melanoma skin cancers and cancers with an excellent prognosis (e.g. e.g. early-stage breast or prostate cancer) • History of cerebrovascular (e.g., ischemic or hemorrhagic stroke, carotid endarterectomy) or cardiovascular accidents (e.g., myocardial infarction, coronary acute syndrome, heart bypass surgery) within the last six months • NYHA Class IV heart failure • Respiratory insufficiency requiring regular supplemental oxygen • Severe chronic kidney disease (e.g. stage V or dialysis or eGFR < 30 ml/min/1,72m^2^) • Decompensated cirrhosis • Alcohol or substances abuse • Inability or unwillingness to provide informed consent • Participation in other trials.

*ADL* Activity of Daily Living, *T2DM* type 2 diabetes mellitus, *eGFR* estimated glomerular filtration rate, *ES* equivalent score, *MoCA* Montreal Cognitive Assessment, *NYHA* New York Heart Association, *SPPB* Short Physical Performance Battery

### Recruitment and randomization

Trained researchers embedded in each diabetology outpatient unit will assess patients for inclusion and exclusion criteria. Those meeting the criteria will be invited to participate and asked to provide written informed consent. After the baseline assessment, participants will be randomly assigned to either the intervention or control group. A secure internet-based system will be used for randomization, assigning participants to the groups in a 1:1 allocation ratio, stratified by center. A total of 60 patients per center will be recruited, for a combined total of 180 participants.

Given the low-risk nature of the intervention, no data monitoring committee has been established for this trial. This approach has been approved by the Ethics Committees at all participating centers.

### Assessment and data collection

Table 2 reports the Gantt charts detailing the timeline and the specialists responsible for the study procedures.

**Table 2:** Gantt chart of timeline and specialists

	Recruitment	Baseline	6th months	12th months
Informed consent	○○○○			
Eligibility criteria	○○○○			
Comprehensive Geriatric Assessment		○○○○	○○○○	○○○○
Diabetologist assessment		●●●●	●●●●	●●●●
Randomization		○○○○		
CGA-driven intervention		○○○○	○○○○	○○○○

Legend: ○○○○ Geriatrician, ●●●● Diabetologist

### Baseline assessment

At baseline, all eligible patients will undergo both diabetologist and geriatric assessments. Demographic, clinical, functional, and nutritional data will be collected through CGA by a geriatric researcher at each center, using validated tools [13].

Collected demographic and clinical data are listed in Table 3.

**Table 3:** Collected demographical and clinical data

**Demographic** - Age - Sex - Ethnicity - Education - Marital status - Living status - Social support - Economic status	**Clinical** - Height - Weight - Body mass index - Waist circumference - Hip circumference - Brachial blood pressure - Ankle blood pressure - Heart rate - Neurological examination - Peripheral neuro-sensory deficits - Diabetic ulcer - Macrovascular diabetes-related complications - Microvascular diabetes-related complications	**Blood tests** - Complete blood count - Glucose - Hemoglobin A1c - Liver function parameters - Renal function parameters - Albumin - Total proteins - Uric acid

Functional data includes Katz’s Activity of Daily Living (ADL) [14] and Lawton-Brody’s Instrumental Activity of Daily Living (IADL) [15]. Physical performance will be assessed through the Short Physical Performance Battery (SPPB) [11], which evaluates (a) standing balance in three different positions, (b) lower limb strength based on the time taken to stand up from a chair five times as quickly as possible, and (c) gait speed over a distance of 4 m at a normal pace. The SPPB total score ranges from 0 (disability/very poor performance) to 12 points (good performance) [16]. Additionally, upper limb strength will be measured through the grip strength using a held-held dynamometer.

Comorbidity will be assessed using the Charlson Comorbidity Index (CCI) [17]. The Snellen Chart test, the Whispered-voice test, and self-reported screening questions will be used to assess visual and hearing impairment, respectively [18]. Additionally, information on medication use will be obtained through clinical interviews.

Cognitive function will be evaluated using the Italian version of the Montreal Cognitive Assessment (MoCA), with appropriate adjustment of raw score [19, 20]. The MoCA is widely regarded as one of the most effective tools for assessing cognitive function in individuals with normal cognition or mild cognitive impairment. Additionally, depression and anxiety will be screened with a 15-item Geriatric Depression scale (GDS) [21] and 10-item Geriatric Anxiety scale (GAS) [22], respectively.

Nutritional status will be evaluated using the Mini-Nutritional Assessment short form (MNA-sf) and bioelectrical impedance analysis (BIA), while dietary habits through the Mediterranean Score Diet (MSD) [23–25].

The level of frailty will be evaluated using the Primary Care Frailty Index (PC-FI) and the Clinical Frailty Scale (CFS) [26, 27].

Lastly, the quality of life will be evaluated with the 12-item Short Form Health Survey (SF-12) [28].

### Interventions

Every recruited patient will receive standard care in accordance with current diabetes guidelines from a diabetologist. Additionally, a trained geriatric researcher will administer the CGA to patients in both the control and the intervention groups. For the intervention group, the CGA will be used to identify and optimize medical treatment concerning functional outcomes and PROMs. Specifically, the CGA will guide medication review and deprescribing, functional training, nutritional advice, and environmental adaptation. Specialist consultation will be recommended for hearing or visual impairments, and cognitive dysfunction (MoCA ES < 4), or in presence of anxiety or depressive symptoms. Patients in the control group will continue to receive standard care as per current guidelines for patients with T2DM, without additional interventions.

### Follow-up

Follow-up visits will be scheduled 6 and 12 months after randomization and conducted at the diabetology outpatient clinic. For patients unable to attend the outpatient clinics due to incapacity or institutionalization, telephone interviews will be conducted with a relative or caregiver present. For hospitalized patients, an outpatient evaluation will be scheduled post-discharge.

During the in-person follow-up evaluations, data collection will include: height, weight, body mass index (BMI), waist and hip circumferences, brachial and ankle blood pressure, heart rate, peripheral neuro-sensory deficits, diabetic ulcer, macro- and micro-vascular diabetes-related complications, recent blood test results, CCI and pharmacotherapy, ADL and IADL, MoCA, SPPB, handgrip, MNA-sf, BIA, MSD, CFS, PC-FI, 15-item GDS and 10-item GAS, SF-12, social support, and living situation, fall occurrences, and hospitalization rates. During telephone interviews, the following data will be collected: self-reported weight, home-measured brachial blood pressure, recent blood test results, CCI and pharmacotherapy, MNA-sf, ADL and IADL, 15-item GDS and 10-item GAS, social support, and living situation, fall occurrences, and hospitalization rates. Lastly, follow-up assessment will include patients’ experience with care, including perceived involvement in treatment decision, clarity of information received, coordination among healthcare professionals, and the perceived value of diabetes self-management education.

### Outcomes

The primary outcome will be the change in physical performance at the end of the follow-up, as assessed by the SPPB score, with a comparison between the control and intervention groups. Secondary outcomes will include changes in the performance of individual SPPB items (balance, gait speed, chair-stand test), changes in cognitive function as measured by MoCA scores, and the incidence or progression of ADL disability. Additionally, we will evaluate the incidence of sarcopenia, falls, and institutionalization. Lastly, other secondary outcomes will include hospitalization rates for all-cause and diabetes-related complications, changes in HbA1c levels, the number of days spent at home post-randomization (DAH), accounting for death, hospitalization, and new care home placement, and all-cause mortality rate.

### Sample size and statistical analysis

Using data from a previous observational study in older Italian patients with diabetes [29], we hypothesized a baseline mean SPPB score of 6.9 with a 1.8 standard deviation. Assuming that a difference of 1 SPPB score point is clinically meaningful [30], we estimated that 72 patients in each group would provide a 90% power at a 5% significance level to detect this difference between the intervention and the control group after 12 months from enrolment. To account for a 20% loss to follow-up, including patients who will die during the study follow-up, we planned to enroll 90 patients for each study group, for a total of 180 participants.

Data will be described as mean and standard deviation or median and interquartile range for continuous variables and frequency and percentage for categorical variables. Baseline characteristics will be compared between intervention and control groups using ANOVA for normally distributed continuous variables, nonparametric tests for non-normally distributed continuous variables, and chi-square or Fisher’s exact test for categorical variables.

Changes in SPPB scores (primary outcome) between study groups over follow-up will be assessed using longitudinal data analysis for repeated measures. Differences in secondary binary outcomes between groups will be analyzed using multivariable logistic regression models or Cox proportional hazard models, as appropriate. Changes in secondary continuous outcomes between groups will also be evaluated using longitudinal data analysis for repeated measures. All statistical models will be adjusted for relevant potential confounders, including socio-demographic factors, comorbidities, medications and study center. Confounders will be selected based on their association with the specific outcome being analyzed and consideration of collinearity. Comorbidities and medications will be incorporated into the models either as individual variables - when specific conditions or treatments are expected to influence the outcome - or as aggregated measures (e.g., total number of comorbidities or medications) to reflect overall disease or treatment burden, depending on their distribution and relevance. A two-tailed p-value < 0.05 will be considered statistically significant.

### Patient and public involvement

Patients and the public are not involved in the design and implementation of this trial.

## Ethics and dissemination

This study has been approved by the Ethics Committee of the clinical centers and will be conducted in accordance with the European Union (EU) 2016/679 and 2016/680 directives and guidelines. Data will be collected on the Research Electronic Data Capture platform (REDCap) [31, 32]. Site access will be protected by a personal password to ensure data safety and confidentiality. Passwords will be given to the researchers by a data manager of the Italian Society of Gerontology and Geriatrics (SIGG). Each patient will be assigned a unique alphanumeric code to which all the data will be linked. This code will be recorded and stored on an encrypted file. To ensure patient anonymity, data will be pseudo-anonymized following the standard ISO 25237:2017. The study findings will be disseminated through a peer-reviewed publication and at appropriate conferences and scientific meetings and shared more broadly through collaborations with patients with diabetes and their caregiver association at both local and national levels.

## Discussion

Diabetes and its complications are associated with several negative health outcomes that can significantly impact the quality of life and the functional independence of older patients with T2DM. Historically, treatment goals for these patients have primarily focused on glycemic control and management of associated cardiovascular risk factors (i.e., blood pressure and lipids profile control) to reduce microvascular and macrovascular diabetes-related complications, respectively. Nevertheless, older patients with T2DM are particularly complex because of multimorbidity [33], polypharmacy [34], and emerging diabetes complications, including frailty, cognitive impairment, and physical disability [35]. Therefore, management of these patients requires a multidimensional evaluation assessing the multiple determinants of health and a tailored multidomain plan of care [36]; indeed, an aggressive and standardized approach characterized by a strict metabolic control might result in negative outcomes including, but not limited to, severe hypoglycemia, particularly among the frailest [37].

Only recently has the importance of the CGA in implementing goal-driven interventions emerged [10]. However, it has yet to be fully integrated into routine diabetes care for older adults. As a result, there is no solid evidence supporting the effectiveness of CGA-driven interventions in delaying the development of functional impairments and achieving PROs in older patients with T2DM. This study aims to address this gap by evaluating the impact of CGA on the complex care needs of older patients with T2DM. Additionally, we propose that incorporating functional outcomes and PROMs as treatment goals may enhance the management of these patients, aligning therapeutic strategies more closely with their specific needs and preferences. Specifically, we anticipate that this multidimensional approach will help preserve better function and prevent or delay the development of disability and other important adverse health outcomes compared to the usual diabetic care practices.

Diabetes has already been linked to reduced muscle performance, which contributes to walking and functional limitations [29]. Furthermore, poor lower-extremity performance has been associated with several critical clinical endpoints in community dwellers and hospitalized patients [11, 38–40]. Therefore, the functional measure adopted as the primary outcome of the present study could serve not only as a treatment goal but also as a strong indicator of the patient’s quality of life. For this purpose, we selected the SPPB, which is easy and quickly measurable in clinical practice and has already been integrated into the PROMs [41]. Simultaneously, the CGA will contribute to the assessment and optimization of other domains (e.g., cognition, physical and psychosocial health, social-environmental circumstances, and polypharmacy), which may contribute to reducing adverse health outcomes and improving satisfaction among patients and caregivers along the care pathway. To comprehensively assess the effect of CGA-driven care pathway, we incorporated a set of secondary outcomes to capture both physical performance-related events, such as the incidence of sarcopenia, falls and institutionalization, and broader clinical endpoints including HbA1c variations, hospitalization rates, mortality and DAH. These measures aim to explore the wider impact of the intervention on the health status and healthcare utilization in this population. Additionally, patient-reported experiences related to involvement in care and support received will be evaluated to provide insight into the intervention’s impact on care quality and patient satisfaction.

This study has some limitations. Implementation of certain aspects of the intervention will depend on patient initiative, as adherence will not be actively monitored between study visits, and specialist consultations will not be scheduled by the study team. As a result, adherence may vary, potentially affecting the consistency of the intervention. To address this, adherence will be assessed using self-reported data collected during follow-up visits. While this approach is practical, it may be subject to recall bias and reporting inaccuracies, which should be taken into account when interpreting the intervention’s potential impact. Lastly, although the study is adequately powered to detect statistically significant effects for the primary outcome, the secondary outcomes were not included in the power calculations. Therefore, analyses of these secondary outcomes should be regarded as exploratory and hypothesis-generating. This limitation may affect the robustness of findings related to secondary outcomes, warranting cautious interpretation.

This study aims to contribute to cumulating evidence concerning the impact of CGA on patients with T2DM. It will reinforce the routine application of an integrated, multidimensional, and multi-professional approach to individual care planning, thereby strengthening the role of patients and addressing their needs to foster a more personalized, inclusive, and participative form of medicine.

In conclusion, the potential impact of this study may extend to improvements in disease management and patient and caregiver satisfaction, as well as to promoting organizational changes. If successful, our approach could inform changes in the practice of diabetologists by incorporating the functional measures outlined and establishing closer collaboration with geriatricians. In the long-term, these changes may help mitigate functional decline and disability progression in older patients with T2DM, with possible positive economic implications on the healthcare system.

## Supplementary Information

Supplementary Material 1.

## Data Availability

No datasets were generated or analysed during the current study.

## Abbreviations

ADA: American Diabetes Association.
ADL: Activity of daily living.
CCI: Charlson Comorbidity Index.
CFS: Clinical Frailty Scale.
CGA: Comprehensive Geriatric Assessment.
T2DM: Type 2 diabetes mellitus.
EU: European Union.
GAS: Geriatric Anxiety Scale.
GDS: Geriatric Depression Scale.
IADL: Instrumental Activities of Daily Living.
MNA-sf: Mini Nutritional Assessment short form.
MoCA: Montreal Cognitive Assessment.
MSD: Mediterranean Score Diet.
NYHA: New York Heart Association.
PC-FI: Primary Care Frailty Index.
PREMs: Patient Reported Experience Measures.
PROs: Patient Reported Outcomes.
PROMs: Patient Reported Outcome Measures.
REDCap: Research Electronic Data Capture platform.
SIGG: Italian Society of Gerontology and Geriatrics.
SF-12: 12-item Short Form Health Survey.
SPPB: Short Physical Performance Battery.
